# The Physiology of Recreation

**Published:** 1895-08-17

**Authors:** 


### The Hospital

An Institutional Journal of

The Medical Sciences and Hospital Administration, with

AN EXTRA NURSING SUPPLEMENT.

Vol. XVIII.—No. 464.] [Aug. 17, 1895.

### The Physiology of Recreation.

Every subject which may properly be called "physiological" belongs naturally to the province of medicine. In the July number of the *Contemporary Review* Mr. Charles Roberts discusses "The Physiology of Recreation." This is eminently a "doctors' question," and a few moments will be well spent in its consideration.

A third part of the time, even of the working classes, Mr. Roberts tells us, is available for recreation. Such a statement demands proof, and this is supplied: "If we include Sundays, Bank Holidays, Christmas Day, and Good Friday, with the half-day weekly holiday now so universal, we make up nine weeks of the year When to these are added the five daily hours which intervene between the close of the working day and the commencement of the night's sleep a further period of nine weeks is completed, making together eighteen weeks, or rather more than a third of the whole year." Practically all other classes, if we except the members of the medical profession, have at least as much leisure as the "industrial classes," ordinarily so called. It is obvious, therefore, that the question of recreation is one of first-rate importance.

Recreation should begin in the cradle. "A baby's life should be all recreation," Mr. Roberts tells us; and on this point we shall most certainly all agree with him. The next proposition may evoke contention. The period of infancy, which includes the first five years of life, is also claimed for recreation. The infant schoolmistress, and, more still, the State, are our opponents here. Nevertheless, Mr. Roberts assures us that infant schools, in an ideal society, would have no existence. As things are at present they may be indispensable; but they are among those serious evils which the improved social condition that we expect science to gradually bring about for us will disestablish and destroy.

Of more immediately practical importance is the question of recreation for young people of both sexes who are not yet fully developed, and for those persons between the ages of twenty and sixty, upon whom the full strain of the world's thought and work presses so urgently and unremittingly. Here we grapple with a problem which every one of us is imperatively called upon to solve. If we solve it scientifically we may say with Mr. Micawber that the result will be "happiness"; if, on the other hand, we solve it foolishly, the result will as certainly be "misery."

In a short article like the present there can be no details. If, however, a great principle, a principle which is so obviously sound that every intelligent reader will at once agree with it, can be stated and fixed firmly in the mind, like a candle in its socket, a great service will be rendered to national physiology. Such a principle is expressed in the old "saw" with which we have all been familiar from childhood, and which tells us that "a change of work is as good a holiday." Now "a change of work" is by no means always, or even often, "as good as a holiday."

For children and young people it is obvious that "games" are the best of all forms of change: much better, Mr. Roberts insists, than any kind of ordered gymnastics, good and serviceable as these latter may be in their places. In childhood and youth games are the very quintessence and perfection of delightful and thoroughly scientific recreation. Two facts strike us here, and demand consideration. Our children, at any rate our town children, no longer play. "The saddest feature of our modern social condition and overcrowding in towns," says Mr. Roberts, "is that children have forgotten how to play." That is a condition of things which science, and especially the science of public medicine, must remedy, or very evil days are in store for us all in the near future.

The reader of mature life, who is in the midstream of nineteenth century stress and strain, and who is so often conscious that recreation is his one resource against permanent breakdown, is perhaps thinking that he is to be forgotten in this brief article. By no means! For him, as for younger people, recreation should always mean "change," and, as far as possible, this change should always include "games." Here the middle-aged Scotchman has a great advantage over his southern fellow countrymen in his universal enthusiasm for the game of "golf." Mr. Roberts names "golf" as one of the typical games for those who are no longer in their first youth. Riding, walking, fishing, shooting are, of course, other well-known varieties of recreation, almost all equally good. What we desire to insist upon is, that if the work of life is to be carried on happily and successfully recreation must be both constant and abundant; and that the one indispensable condition of true recreation is "change." If this change can also be made to include bright and cheerful "games" for persons of all ages its value as recreation will be absolute.

